# The Value of Programmable Shunt Valves for the Management of Subdural Collections in Patients with Hydrocephalus

**DOI:** 10.1155/2013/461896

**Published:** 2013-12-22

**Authors:** Dimitrios Pachatouridis, George A. Alexiou, Evaggelos Mihos, George Fotakopoulos, Spyridon Voulgaris

**Affiliations:** Department of Neurosurgery, University Hospital of Ioannina, P.O. Box 103, Neohoropoulo, 45500 Ioannina, Greece

## Abstract

*Background*. The aim of the present study was to assess the value of electromagnetic programmable shunt valves for the treatment of subdural collections. *Methods*. Adult patients with hydrocephalus of various causes that were treated with programmable shunt valves during the last ten years were retrospectively studied. In 127 patients, 139 electromagnetic programmable shunt valves were implanted. *Results*. A nontraumatic subdural fluid collection was detected in 12 patients. The treatment of these patients consisted of reprogramming of the valve's opening pressure. In 5 patients small subdural hematomas were detected; 4 of these patients were treated by raising the opening pressure alone and one patient required surgical drainage and change of the pressure setting. Traumatic chronic subdural hematomas were detected in 6 patients. These patients were treated by surgical drainage and readjustment of the valve's opening pressure. *Conclusion*. The ability to treat a shunt-related complication, such as a subdural fluid collection, by reprogramming the valve's opening pressure to a higher setting is an advantage over nonprogrammable valves, and it enables the opening pressure to be slowly lowered once the fluid collection is reabsorbed. Based on our results, we believe that programmable shunt valves should be preferred.

## 1. Introduction

Programmable shunt valves have been used for the treatment of hydrocephalus of various causes [1]. Theses shunt systems are prone to complications such as overdrainage similar to nonprogrammable valves [2–4]. When implanting valves of different opening pressure for CSF drainage, several factors must be considered. The most important factor is selecting the optimal valve opening pressure for the individual patient. The selection of the most suitable valve opening pressure preoperatively is very difficult and often pressure adjustments may be required postoperatively [5]. When a nonprogrammable valve is used and there is a shunt-related complication, such as a subdural fluid collection, surgery is required to alter the opening pressure. The aim of the present study was to assess the value of electromagnetic programmable shunt valves for the treatment of subdural collections.

## 2. Material and Methods

### 2.1. Patient Population

Over a 7-year period, 139 electromagnetic programmable shunt valves were implanted in 127 adult patients. There were 68 (53.5%) males and 59 (46.5%) females, mean age 56.7 ± 17.8 years, ranging from 18 to 89 years. In all patients the distal catheter of the shunt was placed in the peritoneal cavity. The opening pressure settings at the time of implantation were selected on the basis of patient's age, diagnosis, and duration of the underlying disease. The initial setting was adjusted while the valve was in its sterile blister package. The underlying conditions that prompted the placement of a shunt valve system are summarized in Table 1.

### 2.2. Shunt Revision

All patients had a minimum 2-year follow-up. During this period, in twelve cases (8.6%) the shunt system was replaced. The replacement was due to shunt infection (25%), blockage by protein clots (25%), proximal catheter obstruction (16.67%), proximal catheter suboptimum position (16.67%), and shunt malfunction, due to difficulty in adjusting the opening pressure (16.67%). Possible causes of adjustment failure were early removal of the programming unit's transmitter and difficulty in accurately positioning the transmitter.

### 2.3. Valve Opening Pressure Adjustments

Adjustments of the valve's opening pressure were made in accordance with clinical or radiological findings obtained during the postoperative period. Changing the polarity of the magnetic field surrounding the valve allows transcutaneous adjustment of the opening pressure, of a programmable valve, in a range from 30 to 200 mm H_2_O in steps of 10 mm H_2_O. This spares the patient from undergoing reoperation to achieve pressure adjustments.

## 3. Results

A nontraumatic subdural fluid collection (hygroma) was detected in 12 (9.4%) patients. The treatment in these cases consisted of reprogramming the valve's opening pressure. No surgery was required in all cases. In 5 patients small subdural hematomas were detected; 4 of these patients were treated by raising the opening pressure alone and one patient required surgical drainage and change of the pressure setting. Traumatic chronic subdural hematomas were detected in 6 patients. These patients were treated by surgical drainage and readjustment of the valve's opening pressure.

## 4. Illustrative Cases

### 4.1. Case 1

**Figure 1:**
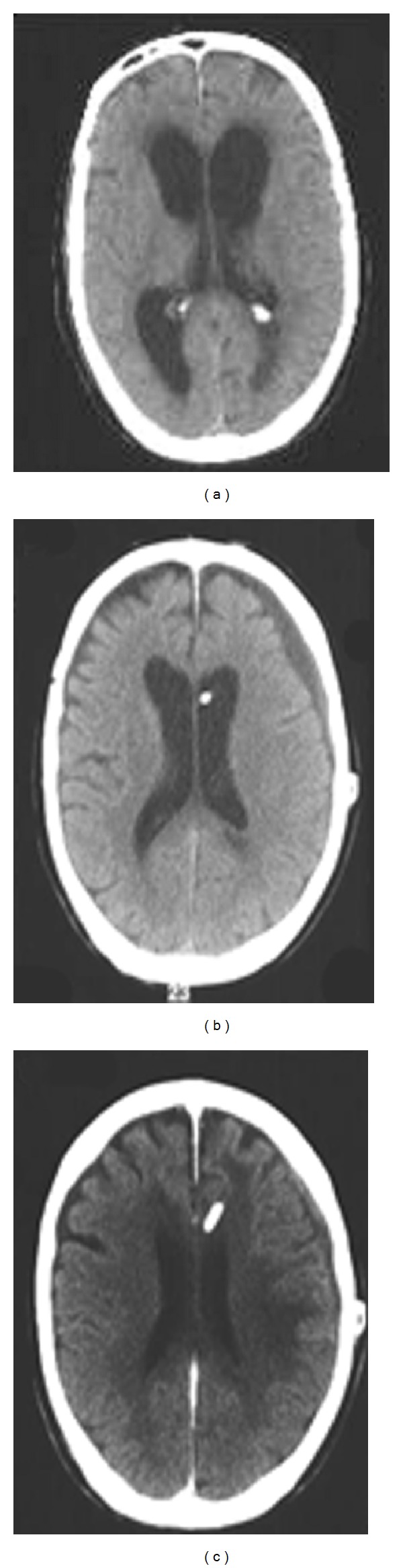
(a) Preoperative CT scan showing enlarged ventricular system. (b) CT scan four weeks after insertion of a shunt, subdural hygroma can be observed (c) CT scan 6 weeks after the adjustment of the opening pressure. The hygroma had reabsorbed.

A 65-year-old patient developed hydrocephalus after treatment of a right cerebelopontine angle tumor (Figure 1(a)). The patient received a programmable valve set at 110 mm H_2_O. Four weeks later a subdural hygroma was detected on follow-up CT (Figure 1(b)). The valve's opening pressure was raised at 130 mm H_2_O. Six weeks after the adjustment the hygroma was resolved (Figure 1(c)).

### 4.2. Case 2

**Figure 2:**
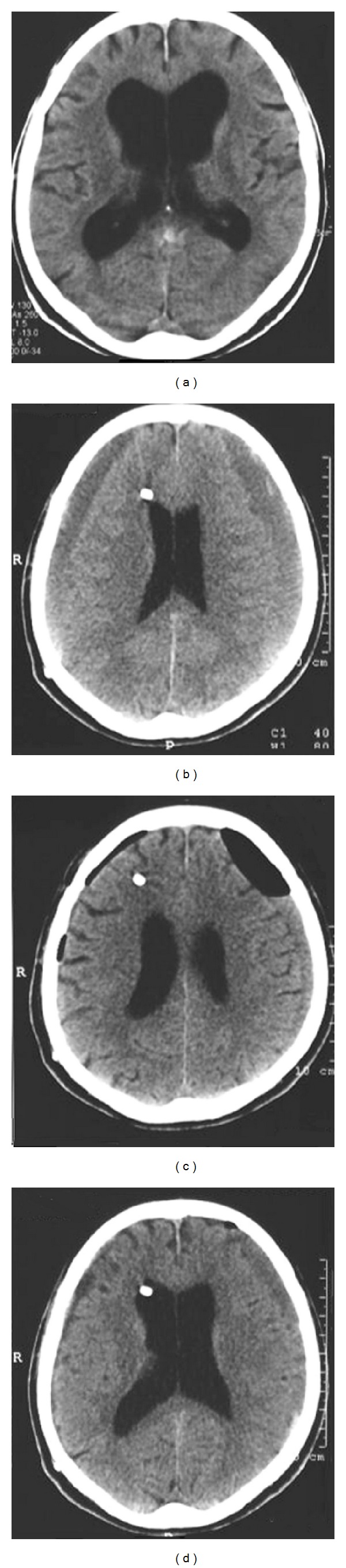
(a) CT scan showing a widened ventricular system. (b) CT scan 35 months after shunt implantation and bilateral traumatic chronic subdural hematomas were demonstrated. (c) Postoperative CT scan 2 days after surgical drainage and adjustment of the opening pressure. (d) CT scan two weeks after the adjustment. The hematomas were almost resolved.

A 73-year-old patient presented with symptoms suggestive of normal pressure hydrocephalus. Brain CT revealed enlarged ventricular system (Figure 2(a)). A programmable valve set at 140 mm H_2_O was placed. Twenty-three months later and 3 months after a minor head injury the patient presented with headache. CT scan was performed and revealed bilateral traumatic chronic subdural hematomas (Figure 2(b)). Bilateral burr holes were performed and the valve's opening pressure was raised at 180 mm H_2_O. Two days after the operation a CT was performed (Figure 2(c)). Two weeks after the operation the subdural hematomas were almost resolved (Figure 2(d)). The valve's opening pressure was lowered at 140 mm H_2_O.

## 5. Discussion

The present study showed that when electromagnetic programmable valves are implanted and a shunt-related complication occurs, the surgeon has the option for a noninvasive reprogramming of the valve's opening pressure. This may be sufficient for the treatment of a subdural collection.

The valve's opening pressure is the most important factor in determining CSF drainage. Subdural effusions occurred in 71% of patients with a low pressure shunt and in 34% of patients with a medium pressure shunt [6]. It is difficult to know at the time of the implantation which patient will need an adjustment later. If the shunt valve's opening pressure is not the optimal one for the patient's intracranial hydrodynamics, complications such as subdural fluid collections may occur during patient's clinical course [7, 8]. Programmable shunt valve proved to be comparable to conventional valves in the overall population of patients with hydrocephalus. Shunt revision rate was similar in both cases [9]. Although no significant difference in the incidence of subdural fluid collections between the programmable and fixed-pressure valve treatment groups was found, a programmable valve gives the surgeon the benefit to adjust the opening pressure setting noninvasively, according to clinical or radiological findings, during the postoperative period [7, 10]. In addition shunt system with programmable valve allows for continued treatment of the patient's known hydrocephalic condition. When subdural fluid collection occurs, the removal or ligation of the shunt to treat this complication can be risky. Patients with programmable valves can in many cases be treated at the outpatient clinic.

Nevertheless, programmable valves are more expensive compared to nonadjustable valves. However, the expenses of CSF shunt valves comprise only a small part when compared with the total cost of treatment and care of patient with hydrocephalus [11]. The lifelong treatment of patients suffering from hydrocephalus mandates the use of shunt valve systems, in which the opening pressure can be adjusted noninvasively. This spares the patient from the physical and psychological stress and the complications that an operation entails. In patients with normal pressure hydrocephalus minor or single larger adjustments to the valve opening pressure can further improve the outcome in those patients who undergo shunting [8]. In patients with a shunt and subdural hematoma, a readjustment of pressure level may be sufficient to reduce hematoma volume [12]. Nevertheless, some patients may suffer delayed subdural hematoma that requires surgery [12]. In our series, all cases with traumatic subdural hematomas were operated and the valve's opening pressure was readjusted.

In conclusion, subdural fluid collections, such as hygromas and hematomas, are common complications in hydrocephalic patients treated with shunt valve systems. When electromagnetic programmable valves are used, the surgeon may noninvasively readjust the valve's opening pressure to a higher setting, if a shunt-related complication occurs. This is an advantage compared to fixed-pressure valves and enables the opening pressure to be slowly lowered once the subdural fluid collection is reabsorbed. Because we cannot predict which patient will need an adjustment later in his lifelong treatment, therefore our suggestion is the usage of programmable shunt valves in all conditions that require CSF drainage.

## Figures and Tables

**Table 1:** Initial diagnosis that prompted shunt placement.

Diagnosis	Number of patients
Aqueductal stenosis	17 (13.4%)
Tumor	11 (8.6%)
Meningitis	7 (5.5%)
Trauma	15 (11.8%)
Intraventricular hemorrhage	8 (6.3%)
Subarachnoid hemorrhage	5 (4%)
Intracerebral hemorrhage	7 (5.5%)
Normal pressure hydrocephalus	57 (44.9%)
